# The Role of LIN28-*let-7*-ARID3B Pathway in Placental Development

**DOI:** 10.3390/ijms21103637

**Published:** 2020-05-21

**Authors:** Asghar Ali, Gerrit J. Bouma, Russell V. Anthony, Quinton A. Winger

**Affiliations:** Department of Biomedical Sciences, Animal Reproduction and Biotechnology Laboratory, 1683 Campus Delivery, Colorado State University, Fort Collins, CO 80523, USA; gerrit.bouma@colostate.edu (G.J.B.); russ.anthony@colostate.edu (R.V.A.); quinton.winger@colostate.edu (Q.A.W.)

**Keywords:** miRNA, trophoblast cells, cell proliferation, ARID3B complex

## Abstract

Placental disorders are a major cause of pregnancy loss in humans, and 40–60% of embryos are lost between fertilization and birth. Successful embryo implantation and placental development requires rapid proliferation, invasion, and migration of trophoblast cells. In recent years, microRNAs (miRNAs) have emerged as key regulators of molecular pathways involved in trophoblast function. A miRNA binds its target mRNA in the 3ʹ-untranslated region (3ʹ-UTR), causing its degradation or translational repression. Lethal-7 (*let-7*) miRNAs induce cell differentiation and reduce cell proliferation by targeting proliferation-associated genes. The oncoprotein LIN28 represses the biogenesis of mature *let-7* miRNAs. Proliferating cells have high LIN28 and low *let-7* miRNAs, whereas differentiating cells have low LIN28 and high *let-7* miRNAs. In placenta, low LIN28 and high *let-7* miRNAs can lead to reduced proliferation of trophoblast cells, resulting in abnormal placental development. In trophoblast cells, *let-7* miRNAs reduce the expression of proliferation factors either directly by binding their mRNA in 3ʹ-UTR or indirectly by targeting the AT-rich interaction domain (ARID)3B complex, a transcription-activating complex comprised of ARID3A, ARID3B, and histone demethylase 4C (KDM4C). In this review, we discuss regulation of trophoblast function by miRNAs, focusing on the role of LIN28-*let-7*-ARID3B pathway in placental development.

## 1. Introduction

Every year, more than 15 million babies are born preterm in the world. A healthy placenta is required for successful establishment of pregnancy and optimal pregnancy outcome [1,2]. Most structural and functional development of the placenta occurs during the first trimester of pregnancy which requires rapid proliferation, invasion, and migration of trophoblast cells [3]. Improper trophoblast function can result in miscarriage, pre-term labor, stillbirth, pre-eclampsia (PE), intrauterine growth restriction (IUGR), and long-term postnatal complication in the mother and fetus [4,5,6,7,8]. The process of rapid trophoblast proliferation and dynamic transformation in placental structure is poorly understood. Previous studies have shown the role of non-coding miRNAs in regulation of trophoblast function. Lethal-7 (*Let-7*) miRNAs are one of the most studied families of miRNAs and have a well-established role in cell proliferation, invasion, migration, differentiation, and metabolism [9,10]. *Let-7* miRNAs reduce cell proliferation by downregulating the proliferation-associated genes [11]. In highly proliferative cells, the RNA binding protein LIN28 represses the production of mature *let-7* miRNAs [12,13]. Low LIN28 and increased *let*-7 miRNAs are thought to be associated with pathogenesis of PE and IUGR [11,14]. During early placental development, dysregulation of miRNAs can lead to reduced proliferation, invasion and migration of trophoblast cells, and contribute to the etiology of placental abnormalities. This review focuses on the role of different miRNAs in trophoblast function, with *let-7* miRNAs being the center of discussion.

## 2. Early Placental Development and Trophoblast Cells

**Figure 1:**
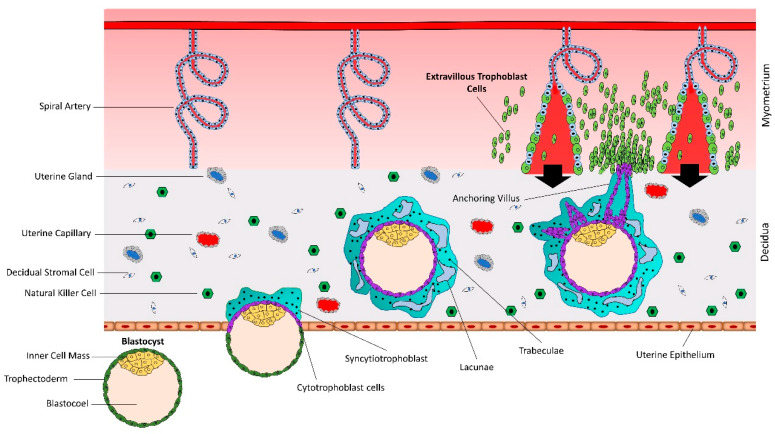
Early placental development and spiral artery remodeling. Human placental development starts with interaction between hatched blastocyst and uterine epithelium. The trophoblast cells that contact with the uterine epithelium transform into highly proliferative cytotrophoblasts (CTBs). Cytotrophoblasts undergo rapid proliferation and some of them fuse to form a multinucleated syncytiotrophoblast (STB). Within a few hours, STB expands and covers whole blastocyst and helps in blastocyst invasion into the uterine decidua. Continuous proliferation of CTBs results in formation of villi. Some CTBs from the tip of anchoring villi break the STB cover, invade the uterine stroma and myometrium, and transform into extravillous trophoblast cells (EVTs). EVTs remodel the spiral arteries to ensure sufficient flow of blood to the placenta.

The human blastocyst is formed at day 4–5 after fertilization and contains an outer most layer of zona pellucida, a single layer of mononuclear trophectoderm or trophoblast (TE), a blastocoel cavity, and an inner cell mass (ICM) or embryoblast. The blastocyst sheds the zona pellucida at day 7, exposing the TE [15]. The hatched blastocyst adheres to the endometrial epithelium, subsequently activating the adherent trophoblast cells to proliferate and give rise to different trophoblast lineages [16] (Figure 1). There are two prominent pathways for trophoblast lineages: syncytial pathway and invasive pathway.

### 2.1. Syncytial Pathway

At the site of attachment, the trophoblast cells transform into rapidly proliferating cytotrophoblasts (CTBs). CTBs undergo rapid proliferation and the newly formed CTBs fuse with each other to form a multinucleated syncytiotrophoblast (STB) [17]. Within a few hours, the STB expands and surrounds the whole blastocyst and mediates the invasion of the blastocyst in the decidualized uterine stroma [18]. The expansion of STB depends upon rapid proliferation and fusion of CTBs [19,20]. By day 21 after conception, the tertiary villi have formed as functional units of the placenta. By week five after conception, the fetoplacental circulation is fully established [21].

### 2.2. Invasive Pathway

Numerous daughter villi arise from tertiary villi, some of which extend to the maternal tissue and are called anchoring or stem villi [22,23,24]. The anchoring sites can be established as early as the second week of gestation [22]. At proximal ends of anchoring villi, some highly proliferating CTBs break free of the overlying STB layer and invade into maternal endometrium and myometrium [24]. As soon as the detached CTBs make contact with decidual extracellular matrix, they differentiate into interstitial extravillous trophoblast cells (iEVTs) [25]. The iEVTs reach the vascular lumen and differentiate into endovascular extravillous trophoblast cells (enEVTs) [26,27]. The enEVTs remodel the spiral arteries which includes loss of endothelial and smooth muscle cells from arterial walls and their replacement by invasive enEVTs, loss of elasticity, dilation of the arterial lumen, and loss of maternal vasomotor control on the remodeled blood vessels [19,27,28,29].

**Figure 2:**
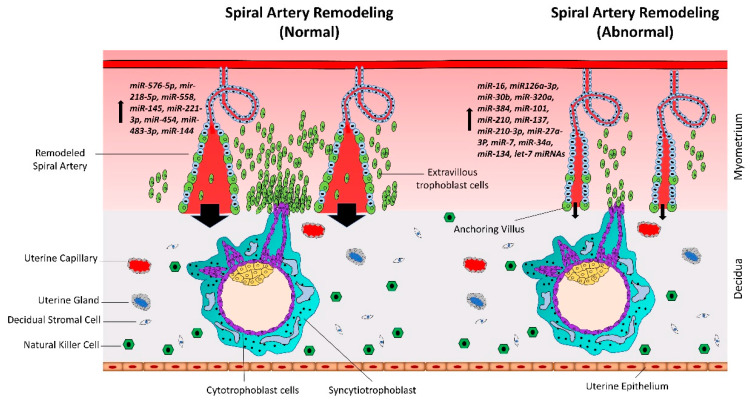
Normal vs. abnormal spiral artery remodeling. CTBs from anchoring villi break out of the SCT layer and enter the uterine stroma where they differentiate into extravillous trophoblasts (EVTs). Spiral artery remodeling is accomplished by invasion and migration of EVTs. EVTs replace the vascular endothelial cells, remodel the spiral arteries, and ensure sufficient flow of blood to the placenta. In placenta-associated disorders like preeclampsia, reduced proliferation of CTBs results in less availability of EVTs. This leads to insufficient remodeling of spiral arteries and reduced blood flow to the placenta. Based on different studies listed in Table 1, a different set of miRNAs is upregulated in trophoblast cells during normal vs. preeclamptic pregnancies.

Spiral artery remodeling is crucial for normal placental development and supplying enough nutrients to the fetus (Figure 2). Inadequate remodeling of the spiral arteries is associated with conditions such as preeclampsia (PE), intrauterine growth restriction (IUGR)/fetal growth restriction (FGR), and recurrent miscarriage, that are harmful for both the mother and the fetus. The proliferation and differentiation of trophoblast cells continues throughout gestation. However, unlike cancerous tissues, the proliferation of trophoblast cells is strictly regulated by complex molecular pathways [30,31]. In recent studies, microRNAs (miRNAs) have been shown to play vital roles in trophoblast proliferation and early placental development.

## 3. Functional Analysis of microRNAs in Trophoblast Cells

MicroRNAs are 20–25 nucleotide-long single stranded RNAs which bind 3ʹ-untranslated region (3ʹ-UTR) of the target mRNA causing its degradation or translational repression [32,33,34,35,36]. MicroRNAs have been shown to play important roles in deciding the fate of trophoblast cells [37]. Proliferation, invasion, and migration of trophoblast cells are critical steps during early human placental development. With increasing evidence for the role of miRNAs in regulation of genes associated with cell proliferation, invasion, and migration, several studies have been conducted to investigate the role of miRNAs in placental development and pathogenesis of placenta-associated disorders.

miRNAs impose their effect by regulating the expression of different genes and the effect of a specific miRNA on the phenotype of a cell or tissue depends upon the role of genes targeted by that miRNA. Hence, depending upon the function of their target genes in trophoblast cells, some miRNAs support successful placental development by promoting trophoblast cell proliferation, invasion and migration, and inhibiting the apoptosis of trophoblast cells, whereas some miRNAs can lead to abnormal placental development by reducing cell proliferation, invasion and migration, and increasing apoptosis of trophoblast cells. Table 1 describes the genes regulated by miRNAs and their effect on functionality of trophoblast cells as described in some recent studies. All gene symbols used in Table 1 are according to the Human Genome Organization (HUGO) Gene Nomenclature.

## 4. Let-7 miRNAs

The lethal-7 (let-7) family of miRNAs was first discovered in 2002 as a development regulator in *Caenorhabditis elegans* [91]. The expression of let-7 miRNAs is low in undifferentiated cells and increases gradually as the cells differentiate during development [92]. Therefore, let-7 miRNAs are also referred to as differentiation-inducing miRNAs. The let-7 mutated *C. elegans* larvae do not mature to the adult stage but keep proliferating and eventually die, earning the name “lethal-7 (let-7)” for this family of miRNAs [91]. Let-7 miRNAs are highly conserved in various animal species [93], suggesting that let-7 miRNAs regulate the same molecular pathways and biological processes in different organisms. In humans, let-7 miRNAs family comprises 12 members including let-7a, let-7b, let-7c, let-7d, let-7e, let-7f, let-7g, let-7i, and miR-98 [94], which originate from eight different genomic loci [95]. Some let-7 miRNAs produced from different genomic loci at different chromosomes have the same sequence. For examples, in humans, let-7a-1, let-7a-2, and let-7a-3 have the same sequence but are encoded by loci on chromosomes 9, 11, and 12, respectively. Similarly, let-7f-1 and let-7f-2 are encoded by different genomic loci but have the same sequence [96]. Let-7 miRNAs have a common seed sequence of seven nucleotides “GAGGUAG” from nucleotide two to eight in all species, which plays an important role in recognizing miRNA response element (MRE) in 3ʹ-UTR of their target mRNA [96]. However, differences in non-seed flanking sequence of let-7 miRNAs affect target specificity [97,98]. Presence of similar seed sequence in all let-7 miRNAs across different species suggests that let-7 miRNAs have the same mechanism for target recognition and might have overlapping targets.

The microarray analysis data from *C. elegans* show that *let-7* miRNAs regulate the expression of thousands of genes, directly and indirectly, indicating their widespread role in biological processes [12]. *Let-7* miRNAs play profound roles in embryo development, glucose metabolism, cell pluripotency and differentiation, tumorigenesis, tissue regeneration, age of onset of puberty and menopause in humans, and organ growth [99,100]. Various studies have shown that *let-7* miRNAs induce cell differentiation and act as fundamental tumor suppressors by downregulating oncogenes [13,101,102,103]. At early stages of cancer development, *let-7* miRNAs are downregulated and *let-7* targeted oncofetal genes (LOG) are re-expressed [104]. Comparative bioinformatics analysis shows that *let-7* miRNAs target several oncofetal genes including high mobility group AT-hook 2 (HMGA2), insulin like growth factor 2 mRNA binding protein 1 (IMP1), IMP2, IMP3, and malignancy marker nucleosome assembly protein 1 like 1 (NAP1L1) [104]. In hematopoietic stem cells, *let-7* miRNAs inhibit transforming growth factor β (TGFβ) pathway and high mobility group AT-hook 2 (HMGA2), decide the fate of these cells, and regulate cell proliferation, self-renewal and differentiation [105,106]. 

*Let-7* miRNAs are synthesized following the same general mechanism for miRNA synthesis. The *let-7* loci are transcribed as pri-*let-7* miRNA, then processed into 67–80 nucleotide long pre-*let-7* miRNA by microprocessor complex [95]. Based on the mechanism of further processing, pre-*let-7* miRNAs are divided in two groups: Group I pre-*let-7* miRNAs (pre-*let-7a-2, 7c*, and *7e*) are processed in cytoplasm by direct action of Dicer, whereas group II pre-*let-7* miRNAs (all remaining *let-7s*) are mono-uridylated prior to processing by Dicer [107]. Action of Dicer produces 22 nucleotide long mature *let-7* miRNAs, called *let-7-5p*. As a part of miRNA induced silencing complex (miRISC), *let-7* miRNAs suppress a wide range of genes involved in development, cell proliferation, metabolism, and other important physiological processes [108]. There is no significant difference in expression of pri-*let-7* and pre-*let-7* miRNAs between undifferentiated and differentiated cells, however mature *let-7* miRNAs are high in differentiated cells compared to undifferentiated cells [109].

Mature *let-7* miRNA is a part of hairpin structure in pri- and pre-*let-7* miRNA. This hairpin structure contains mature *let-7* miRNA (*let-7-5p)* in the stem and a partially complimentary strand of nucleotides called *let-7-3p* miRNA, connected by a terminal loop region of different lengths called pre-element (preE) [110]. The process of generation of mature *let-7* miRNAs is more precisely regulated compared to the synthesis of other miRNAs. Different proteins regulate posttranscriptional biogenesis of mature *let-7* miRNA by binding the preE region of pri- and pre-*let-7* miRNAs [111]. One of the most prominent mechanism for regulation of *let-7* miRNAs biogenesis is through LIN28 [112].

## 5. Suppression of *let-7* miRNAs by LIN28

LIN28 is a highly conserved RNA binding protein with two paralogues, LIN28A and LIN28B. Both LIN28A and LIN28B have a cold-shock domain (CSD) at the N-terminal and two zinc knuckle domains (ZKDs) at the C-terminal [113]. LIN28 promotes cell proliferation and inhibits cell differentiation [114]. LIN28 is also involved in reprogramming of differentiated somatic cells into tumor or stem cells, hence known as oncoprotein [115,116]. Reduced expression of LIN28 in embryos results in reduced prenatal growth and development and long-term metabolic abnormalities [117]. Knockout of LIN28A in mice leads to perinatal lethality while LIN28B knockout results in postnatal growth abnormalities in males. Knockout of both LIN28A and LIN28B in mice is embryonically lethal at around E13. Conditional knockout of LIN28A and LIN28B in mice at six weeks of age does not produce any evident phenotype [118]. Collectively, these findings show that LIN28 has a more profound role during prenatal development and organogenesis. 

LIN28 regulates expression of several genes either directly binding to the mRNA of target genes or by repressing the production of mature *let-7* miRNAs, later being a more prevalent mechanism [118,119]. There are conflicting theories about the localization of LIN28A and LIN28B in cells. LIN28A is predominantly localized in the cytoplasm but can be found in the nucleus as well [120,121]; however, according to another study, LIN28A is exclusively localized in the cytoplasm [122]. LIN28B has a nucleolar and nuclear localization signal, while others found it predominantly in cytoplasm with a possibility to shuttle to the nucleus [113,119,123]. LIN28A and LIN28B selectively repress the expression and maturation of *let-7* miRNAs by distinct mechanisms, without directly affecting the expression of other miRNAs [124]. LIN28A CSD binds GNGAY motif while ZKDs bind GGAG motif in the stem loop pre-*let-7* miRNA in the cytoplasm. After binding to pre-*let-7* miRNA, LIN28A recruits terminal uridylyl transferase (TUTase) Zcchc11 (also referred as TUT4). TUT4 causes polyuridylation of pre-*let-7* miRNA which blocks the cleavage of pre-*let-7* miRNA by Dicer and hence inhibits the production of mature *let-7* miRNAs [124,125,126]. Polyuridylated pre-*let-7* miRNA is recognized and degraded by exonuclease Dis3L2 [127]. The mechanism of *let-7* miRNA suppression by LIN28B remains controversial and there are four different theories. First, LIN28B inhibits maturation of *let-7* miRNAs by TUT4 independent mechanism. In the nucleus, LIN28B binds the pri-*let-7* miRNA by its CSD and ZKDs and inhibits its processing by a microprocessor [122]. Second, in the cytoplasm, LIN28B binds to pre-*let-7* miRNA and inhibits its processing by Dicer [128]. Third, in the cytoplasm, LIN28B binds to pre-*let-7* miRNA and leads to its polyuridylation by recruiting an unknown TUTase, leading to its degradation [121]. Fourth, in the nucleolus, LIN28B has the ability to sequester pri-*let-7* miRNAs and hence inhibits further processing to mature pre-*let-7* miRNAs [122].

In 2018, Ustianenko et al. demonstrated that LIN28 selectively regulates a subclass of *let-7* miRNAs [129]. Using single nucleotide resolution, they identified –(U)GAU- as the new binding motif of the CSD. Some pre-*let-7* miRNAs with both (U)GAU and GGAG motifs in the stem loop make a stronger and stable interaction with LIN28 and are referred to as CSD^+^, while the others which do not contain (U)GAU motif are called CSD^-^. The CSD^+^ subclass includes pre-*let-7b,* pre-*let-7d,* pre-*let-7f-1,* pre-*let-7g,* pre-*let-7i*, and *miR-98.* The CSD^-^ subclass includes pre-*let-7a-1,* pre-*let-7a-2,* pre-*let-7-3,* pre-*let-7c,* pre-*let-7e*, and pre-*let-7f-2* [110,129]. Although all *let-7* miRNAs express ZKD binding GGAG motif, both LIN28A and LIN28B have shown greater binding affinity for CSD^+^ pre-*let-7* miRNAs, hence leading to their polyuridylation and repression [129].

## 6. Gene Regulation by LIN28-*let-7* miRNA Axis in Trophoblast Cells

**Figure 3:**
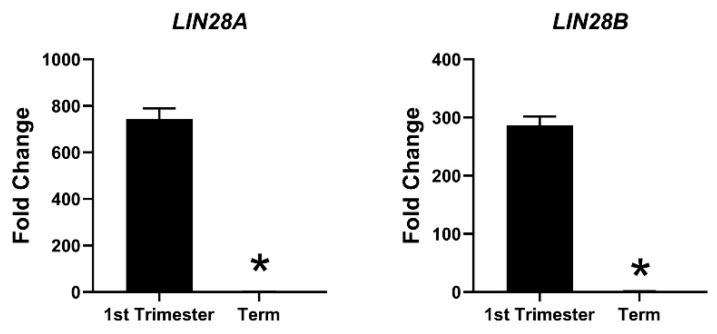
mRNA was extracted from first trimester (week 11) and term human placentas and *LIN28A* and *LIN28B* mRNA levels were measured using real-time RT-PCR, where * *p* < 0.05.

Due to the profound role of *let-7* miRNAs as differentiation-inducing miRNAs, the focus of our lab is to investigate the role of LIN28-*let-7* miRNA axis in trophoblast cells. Both LIN28A and LIN28B are highly expressed in human placenta and are localized to trophoblast cells [11,130,131,132]. High throughput genotyping array reveals that LIN28B is paternally imprinted in human placenta [133,134]. Using single cell transcriptome profiling, Liu et al. identified 14 different cell types in human placenta and showed that paternally imprinted LIN28B has high expression in CTBs, EVTs and STB, whereas it has no to low expression in mesenchymal cells, macrophages, and blood cells in placenta [135]. They further showed that LIN28B expression in week 24 EVTs was lower compared to week 8 EVTs [135], suggesting that expression of LIN28B in trophoblast cells reduces as the pregnancy progresses. LIN28B is the main paralogue of LIN28 in human placenta and *LIN28B* mRNA is 1300-fold higher compared to *LIN28A* mRNA in term human placental tissue [14]. Immunohistochemical analysis of term human placenta shows that LIN28B expression in CTBs and STB is higher compared to placental decidual cells [14]. In 2013, Gu el al. compared the expression of miRNAs between first and third trimester human placentas [136]. They reported that along with many other miRNAs, *let-7a, let-7c, let-7d, let-7f, let-7g*, and *let-7i* are upregulated in third trimester compared to first trimester human placenta [136]. We measured *LIN28A* and *LIN28B* mRNA in first trimester (11 week) vs. term human placentas and found that *LIN28A* mRNA was nearly 700-fold higher and *LIN28B* mRNA was nearly 300-fold higher in first trimester compared to term human placenta (Figure 3). Based on these results, we suggest that increased expression of *let-7* miRNAs in term human placentas, reported by Gu et al., is due to reduced expression of *LIN28A* and *LIN28B*. Low LIN28 and higher level of *let-7* miRNAs in term placenta compared to first trimester placenta suggest that the proliferation rate of trophoblast cells is higher during the first trimester and decreases with advancement in gestational age. As LIN28-*let-7* miRNA axis regulates expression of several genes, it would not be surprising to see a difference in gene expression in first trimester vs. third trimester human placenta.

In IUGR pregnancies, the size of placenta is significantly smaller compared to normal pregnancies [137], which suggests the role of reduced trophoblast proliferation in etiology of IUGR. In a recently published study, we showed that term human placentas from IUGR pregnancies have low LIN28A and LIN28B, and high *let-7* miRNAs compared to term human placentas from normal pregnancies [11]. Canfield et al. reported that term human placentas from preeclamptic pregnancies have reduced LIN28B but no change in LIN28A compared to normal term placentas [14]. They further demonstrated that in first trimester human placenta, LIN28B is higher in extravillous cytotrophoblasts compared to villous trophoblast cells, indicating their role in trophoblast cell invasion [14]. Low LIN28 and high *let-7* miRNAs during the first trimester of pregnancy can lead to reduced trophoblast proliferation and invasion leading to pregnancy-related disorders.

Due to the limitation that humans cannot be used as experimental models, most studies investigating molecular mechanisms involved in human placental development are conducted using placental cell lines. Commonly used human trophoblast-derived cell lines include BeWo, ACH-3P, JEG3, JAR, Sw.71, and HTR8/SVneo. LIN28A knockdown in immortalized first trimester human trophoblast (ACH-3P) cells drives these cells towards syncytial differentiation and increases the expression of syncytiotrophoblast markers including *hCG, LGALS13*, and *ERVW-1* [132]. Moreover, knockdown of LIN28A increases the expression of *let-7* miRNAs including *let-7a, let-7c, let-7d, let-7e, let-7g*, and *let-7i* [132], suggesting that differentiation of cells might be due to increased levels of *let-7* miRNAs. Overexpression of LIN28B in HTR8 cells increases cell proliferation, invasion, and migration, whereas knockdown of LIN28B in JEG3 cells reduces cell proliferation [14]. In a recently published study, we further investigated the correlation between LIN28 and *let-7* miRNAs in trophoblast cells using first trimester human trophoblast-derived ACH-3P and Sw.71 cells. ACH-3P cells were generated by fusing first trimester human trophoblast cells with human choriocarcinoma cells, whereas Sw.71 cells were generated by overexpressing human telomerase reverse transcriptase (h-TERT) in first trimester human trophoblast cells [138,139]. These cell lines have contrasting levels of LIN28 and *let-7* miRNAs [11]. ACH-3P cells have high expression of LIN28A and LIN28B whereas these proteins are not detectable in Sw.71 cells [11]. The expression of all *let-7* miRNAs is 50–500-fold higher in Sw.71 cells compared to ACH-3P cells, potentially due to depleted LIN28A and LIN28B in Sw.71 cells which are major suppressors of *let-7* miRNAs [11]. The contrasting levels of LIN28 and *let-7* miRNAs between ACH-3P and Sw.71 cells are potentially due to the difference of methodology used to generate these cell lines. LIN28A knockout in ACH-3P cells increases *let-7a, let-7b, let-7c, let-7d*, and *let-7e*, whereas LIN28B knockout in ACH-3P cells increases *let-7a, let-7b, let-7c, let-7d, let-7e*, and *let-7i* [11]. According to another study, knockdown of LIN28B in ACH-3P cells increases *let-7c, let-7d, let-7e, let-7f*, and *let-7i* [140]. Double knockout of LIN28A and LIN28B in ACH-3P cells results in increased expression of all *let-7* miRNAs compared to knockout of either LIN28A or LIN28B [11]. Similarly, LIN28A overexpression in Sw.71 cells decreases *let-7d* and *let-7i*, whereas LIN28B overexpression causes reduction in all *let-7* miRNAs. However, overexpression of both LIN28A and LIN28B in Sw.71 cells results in decreased expression of all *let-7* miRNAs compared to overexpression of either LIN28A or LIN28B [11]. These results suggest that LIN28A and LIN28B work in coordination to suppress *let-7* miRNAs and manipulating one paralogue of LIN28 in human trophoblast cells might not induce a similar phenotype compared to if both paralogues are changed.

The majority of *let-7*-regulated genes are associated with cell proliferation, migration, and invasion—processes which are crucial during early human placental development. We recently demonstrated that double knockout of LIN28A and LIN28B in ACH-3P cells increases in *let-7* miRNAs and leads to reduction in expression of proliferation-associated genes including high-mobility group AT-hook 1 (*HMGA1*), MYC protooncogene (*c-MYC*), vascular endothelial growth factor A (*VEGF-A*), and Wnt family member 1 (*WNT1*). LIN28A/B knockout reduces trophoblast cell proliferation and drives them towards differentiating to a syncytiotrophoblast [11,130]. Similarly, double knockin of LIN28A/B in Sw.71 cells leads to reduction in *let-7* miRNAs and increases the expression of *HMGA1*, *c-MYC*, *VEGF-A*, and *WNT1* [11]. Other than its role in cell proliferation, VEGF-A is required at all steps of angiogenesis during placental development [21]. Reduced expression of VEGF-A due to high *let-7* miRNAs can lead to serious pregnancy complications due to impaired angiogenesis in placenta.

Several studies have demonstrated that *let-7* miRNAs bind the 3ʹ-UTR of HMGA2 and reduce its expression in cancer cells [102,141,142]. However, in a recent study, we found a different mechanism of HMGA2 regulation in human trophoblast cells. Double knockout of LIN28A/B in ACH-3P cells increases *let-7* miRNAs but does not change HMGA2 expression [130]. Along with increased *let-7* miRNAs, LIN28A/B double knockout also increases miR-182. The exact mechanism behind increases in miR-182 in LIN28A/B double knockout ACH-3P cells is not clear. We further showed that HMGA2 expression in trophoblast cells is regulated by a transcription-repressing complex comprised of breast cancer susceptibility gene 1 (BRCA1), CtBP-interacting protein (CtIP), and zinc finger protein 350 (ZNF350). This complex, also called BRCA1 repressor complex, binds the promoter region of HMGA2 and inhibits its transcription [130]. In LIN28A/B double knockout ACH-3P cells, high miR-182 targets BRCA1 leading to inhibition of BRCA1 repressor complex and hence increases HMGA2 expression [130]. Therefore, the expected decrease in HMGA2 due to high *let-7* miRNAs is rescued by inhibition of BRCA1 repressor complex. These findings indicate that all genetic pathways demonstrated in the cancer cells might not be applicable in trophoblast cells. It further suggests that rapid proliferation of trophoblast cells during early placental development is more precisely regulated compared to cancer cells.

Although in vitro studies demonstrate the vital role of LIN28-*let-7* miRNA axis in trophoblast function, its role in placental development in vivo is not well understood. Using sheep as an experimental model, we investigated the role of LIN28-*let-7* miRNA axis in trophoblast proliferation in vivo. In sheep, the hatched blastocyst undergoes a phase of trophectoderm elongation before attachment to the uterine epithelium. The conceptus elongation is accomplished by rapid proliferation of trophoblast cells. Trophoblast proliferation is a critical process in early placental development both in humans and sheep. Trophoblast specific knockdown of LIN28A or LIN28B in sheep leads to reduced conceptus elongation due to reduced proliferation of trophoblast cells [143], suggesting that both LIN28A and LIN28B are equally important in early placental development. Knockdown of LIN28A and LIN28B leads to an increase in *let-7* miRNAs and decrease in expression of proliferation-associated genes including insulin like growth factor 2 mRNA binding proteins (IGF2BP1-3), high mobility group AT-hook 1 (*HMGA1*), AT-rich interaction domain 3B (ARID3B), and MYC protooncogene (*c-MYC*) [143]. Additionally, overexpression of LIN28A or LIN28B in immortalized ovine trophoblast cells (iOTR) reduces *let-7* miRNAs, increases the expression of proliferation associated genes, and increases cell proliferation [143]. These findings further strengthen the data from in vitro studies about the role of LIN28-*let-7* miRNA axis in proliferation of human trophoblast cells.

## 7. LIN28-*let-7*-ARID3B Pathway in Trophoblast Cells

AT-rich interactive domain (ARID) proteins, first recognized in 1997, are a family of 15 proteins which binds to AT-rich regions of DNA [144,145]. ARID proteins play an important role in cell proliferation, differentiation, and development, and are upregulated in tumorous tissues [146]. The subfamilies of ARID proteins include ARID1, ARID2, ARID3, ARID4, ARID5, JARID1, and JARID2. The ARID3 subfamily has three members including ARID3A, ARID3B, and ARID3C. Although most of the ARID proteins act as tumor suppressors, ARID3A and ARID3B promote tumorigenesis [144]. ARID3A inhibits cell differentiation, promotes cell proliferation, and increases survival potential of cells [146,147], whereas ARID3B promotes proliferation, invasion, and migration of cancer cells [148,149,150]. However, ARID3B is more widely expressed in different tissues compared to ARID3A, suggesting more involvement of ARID3B in biological functions [145]. ARID3A and ARID3B are structurally similar and bind a similar region of DNA. Both ARID3A and ARID3B have an extended central ARID domain and two conserved amino acid domains at the C-terminal, termed REKLES α and REKLES β. Only members of the ARID3 subfamily have REKLES domains [151].

**Figure 4:**
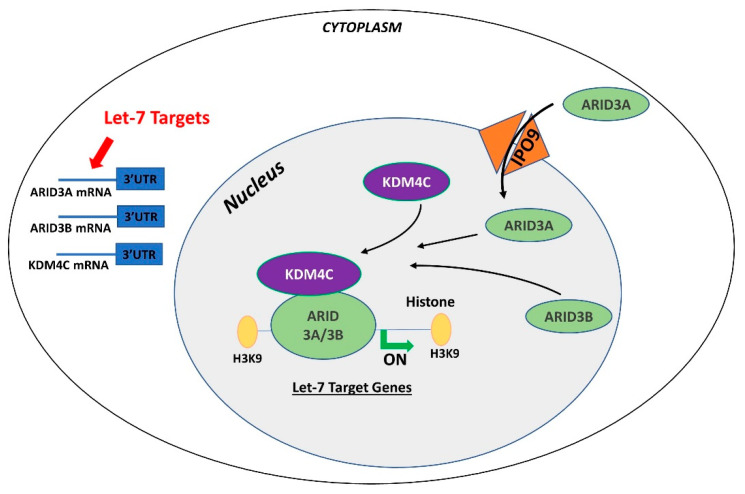
Gene regulation by the AT-rich interactive domain (ARID)3B complex. ARID3A is imported in the nucleus by importin 9 (IPO9), where it binds ARID3B and histone demethylase 4C (KDM4C) to form the ARID3B complex. The ARID3B complex binds in the promoter regions and activates transcription of *let-7* target genes. Other than directly binding the mRNA of their target genes, *let-7* miRNAs also target the ARID3B complex and reduce its expression, ultimately leading to reduced expression of *let-7* target genes.

In contrast to ARID3B which is exclusively localized in the nucleus, ARID3A shuttles between the nucleus and cytoplasm. Once in the nucleus, ARID3A interacts with ARID3B through REKLES β domain. Therefore localization of ARID3A in the nucleus is dependent on its interaction with ARID3B in the nucleus, suggesting the dominant role of ARID3B [151,152]. In cancer cells, ARID3A and ARID3B recruit histone demethylase 4C (KDM4C) to make a tri-protein complex, called the ARID3B complex [153]. The ARID3B complex binds in the promoter areas of stemness genes and *let-7* target genes, leading to histone demethylation by KDM4C and increased gene expression by initiation of transcription [153]. Therefore, genes regulated by the ARID3B complex also include *let-7* miRNA target genes. Liao et al. further demonstrated that both ARID3A and ARID3B are targeted by *let-7* miRNAs. Hence, other than directly targeting the mRNAs of target genes, *let-7* miRNA can indirectly regulate their target genes by targeting and reducing the expression of *ARID3A* and *ARID3B* (Figure 4) [11,153].

Both ARID3A and ARID3B have high expression in human trophoblast cells [154,155]. ARID3A knockout mice have severe structural defects in placenta [156]. In ACH-3P cells, ARID3A, ARID3B, and KDM4C make the tri-protein ARID3B complex [11]. In term human placentas from IUGR pregnancies, LIN28A and LIN28B are low, *let-7* miRNAs are high, and ARID3A and ARID3B are low, which suggest a correlation between LIN28-*let-7* miRNA axis and the ARID3B complex [11]. Due to the well-established pathway of regulation of *let-7* target genes through ARID3B complex and their role in cell proliferation, it is important to understand this phenomenon in early placental development. We recently showed the correlation between LIN28-*let-7* miRNA axis and the ARID3B complex using ACH-3P and Sw.71 cells. Double knockout of LIN28A and LIN28B in ACH-3P cells increases *let-7* miRNAs and decreases the expression ARID3A, ARID3B, and KDM4C. Similarly, double knockin of LIN28A and LIN28B in Sw.71 cells decreases *let-7* miRNAs and increases expression of ARID3A, ARID3B, and KDM4C [11]. In trophoblast cells, the ARID3B complex binds to the promoter areas of proliferation-associated *let-7* target genes including *HMGA1, c-MYC, VEGF-A*, and *WNT1,* facilitating their transcription via KDM4C mediated histone demethylation. ARID3B knockout ACH-3P cells and KDM4C cannot be recruited in the promoter regions of *HMGA1, c-MYC, VEGF-A* and *WNT1*, and expression of these genes is also significantly reduced [73]. Moreover, ARID3B knockout ACH-3P cells have a reduced proliferation rate compared to control cells [11]. Knockdown of LIN28A or LIN28B in sheep trophectoderm increases *let-7* miRNAs and reduces the expression of ARID3A and ARID3B [143], showing regulation of the ARID3B complex by LIN28-*let-7* miRNA axis in vivo. Collectively these findings show that *let-7* miRNAs target the ARID3B complex in trophoblast cells, and the ARID3B complex regulates genes with known importance in placental development.

## 8. Conclusions

**Figure 5:**
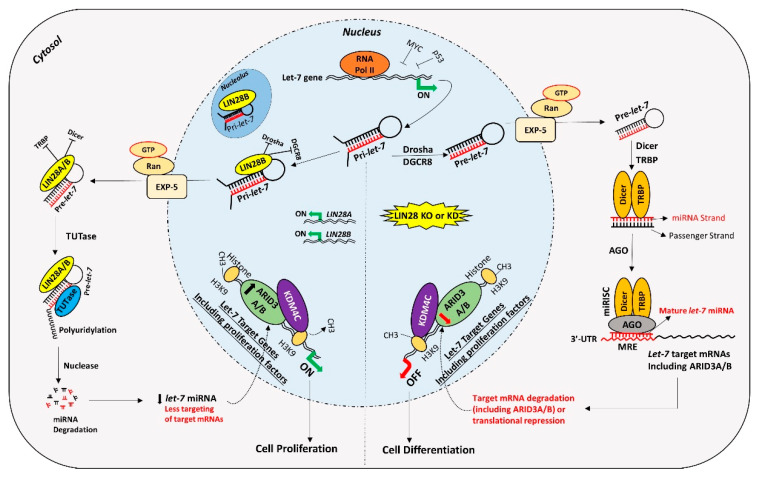
Proposed mechanism for gene regulation in trophoblast cells. Left panel of figure: LIN28 represses the biogenesis of mature *let-7* miRNAs by binding pri-*let-7* and pre-*let-7* miRNAs and inhibiting their processing. Due to the low level of mature *let-7* miRNAs in the cells, there will be less targeting of proliferation-associated genes. Moreover, the ARID3B complex will initiate the transcription of proliferation-associated genes and increase their expression. Increased expression of proliferation-associated genes will lead to increased cell proliferation. Right panel of figure: If LIN28 is knocked-out or knocked-down, there will be no suppression of *let-7* miRNA biogenesis. High *let-7* miRNAs will target and reduce the expression of proliferation associated genes and the ARDI3B complex, driving the cells towards differentiation.

For a long time, transcription activating proteins were thought to be the main regulators of gene expression in cells. However, in recent years microRNAs have emerged as “regulators of the regulators”. miRNAs regulate important processes in trophoblast cells including cell proliferation, differentiation, invasion, and migration. Although identification of widespread *let-7* miRNA target genes makes them an important player in placental development, the role of other miRNAs and molecular pathways in placental development cannot be ignored. Although different studies have reported reduced LIN28 and high *let-7* miRNAs in term human placentas from preeclamptic and IUGR pregnancies, the exact cause of this dysregulation is not clear. Epigenetic modifications due to adverse uterine environment, random genetic mutations, prenatal insults like hypoxia, oxidative stress, maternal malnutrition, and gestational stress are some of the possible factors which can dysregulate LIN28-*let-7* axis in trophoblast cells. LIN28-*let-7*-ARID3B pathway regulates trophoblast cell proliferation by modulating the expression of proliferation associated genes in vitro and in vivo. The trophoblast cells with low LIN28 will have high *let-7* miRNAs and low ARID3B. There are two possible pathways of regulation of proliferation-associated genes by *let-7* miRNAs in trophoblast cells (Figure 5). One pathway involves binding of *let-7* miRNAs in 3ʹ-UTR of their target mRNA leading to mRNA degradation or translational repression. Secondly, *let-7* miRNAs target ARID3A, ARID3B, and KDM4C to inhibit or reduce transcriptional activation of proliferation-associated genes by the ARID3B complex. Therefore, trophoblast cells with high *let-7* miRNAs will have reduced expression of proliferation factors and more of a tendency to differentiate. High *let-7* miRNAs during early placental development reduce cell proliferation, invasion, and migration of trophoblast cells, leading to placental abnormalities. *Let-7* miRNAs might be the major players in pathogenesis of placenta-associated disorders. miRNAs can be readily measured in peripheral blood, tissue biopsies, saliva, cerebrospinal fluid, urine, and other biological samples. High *let-7* miRNAs are upregulated in IUGR and preeclamptic placentas, suggesting that *let-7* miRNAs can be potential biomarkers for early diagnosis of PE and IUGR. It remains to be explored if early stage placentas from compromised pregnancies will have high *let-7* miRNAs and will the increase in *let-7* miRNAs in placenta be reflected in maternal blood. Animal models of IUGR can be used to answer these questions.

## Figures and Tables

**Table 1:** Gene regulation by miRNAs in trophoblast cells.

miRNA	Target Genes	Reference	Effect of Higher miRNA Expression
*let-7*	*ARID3A*, *ARID3B*, *HMGA1*, *cMYC*	[11]	Reduces proliferation and invasiveness of trophoblast cells
*miR-384*	*STAT3*	[38]	
*miR-106b*	*MMP-2*	[39]	
*miR-203*	*VEGFA*	[40]	
*miR-520g*	*MMP-2*	[41]	
*miR-210*	*Notch1*	[42]	
*miR-16*	*Notch2*	[43]	
*miR-320a*	*IL-4*	[44]	
*miR-320a*	*ERRγ*	[45]	
*miR-210-3p*	*FGF1*	[46]	
*miR-7*	*EMT-related TFs*	[47]	Reduces migration and invasion of trophoblast cells
*miR-218*	*SOX4*	[48]	
*miR-34a-5p*	*Smad4*	[49]	
*miR-193b-3p*	*TGF-β2*	[50]	
*miR-34a*	*MYC*	[51]	
*miR-519d*	*MMP-2*	[52]	
*miR-101*	*CXCL6*	[53]	
*miR-34a*	*Notch*	[54]	
*miR-431*	*ZEB1*	[55]	
*miR-145-5p*	*Cyr61*	[56]	
*miR-134*	*ITGB1*	[57]	
*miR-27a-3p*	*USP25*	[58]	
*miR-362-3p*	*Pax3*	[59]	
*miR-181a-5p*	*IGF2BP2*	[60]	
*miR-137*	*FNDC5*	[61]	
*miR-30b*	*MXRA5*	[62]	
*miR-30a-3p*	*IGF1*	[63]	
*miR-18b*	*HIF-1α*	[64]	
*miR-299*	*HDAc2*	[65]	
*miR-454*	*ALK7*	[66]	Increases proliferation of trophoblast cells
*miR-145*	*MUC1*	([67]	
*miR-221-3p*	*THBS2*	[68]	
*miR-126a-3p*	*ADAM9*	[69]	
*miR-483-3p*	*RB1CC1*	[70]	
*miR-144*	*PTEN*	[71]	
*miR-518b*	*Rap1b*	[72]	
*miR-218-5p*	*TGFB2*	[73]	Promotes endovascular extravillous trophoblast cells (enEVTs) and spiral artery remodeling
*miR-210*	*CPEB2*	[74]	Inhibits trophoblast syncytialization
*miR-106a*	*hCYP19A1, hGCM1*	[75]	
*miR-558*	*TIMP4*	[76]	Enhances invasion of trophoblast cells
*miR-576-5p*	*TFAP2A*	[77]	
*miR-184*	*WIG1*	[78]	Promotes apoptosis of trophoblast cells
*miR-133*	*Rho/ROCK*	[79]	
*miR-152*	*Bax, Bcl-2*	[80]	
*miR-155*	*HIF-1α*	[81]	
*miR-371a-5p*	*XIAP*	[82]	
*miR-520*	*PARP1*	[83]	
*miR-34a*	*BCL-2*	[84]	
*miR-18a*	*ER1*	[85]	
*miR-182*	*BRCA1*	[86]	
*miR-23a*	*XIAP*	[87]	
*miR-101-3p*	*mTOR*	[88]	
*miR-96-5p*	*mTOR, Bcl-2*	[88]	
*miR-200c*	*Wnt/β-catenin*	[89]	
*miR-125a*	*MCL1*	[90]	
